# Discovery and Characterization of Two Novel Salt-Tolerance Genes in *Puccinellia tenuiflora*

**DOI:** 10.3390/ijms150916469

**Published:** 2014-09-18

**Authors:** Ying Li, Tetsuo Takano, Shenkui Liu

**Affiliations:** 1 Key Laboratory of Saline-alkali Vegetation Ecology Restoration in Oil Field (SAVER), Ministry of Education, Alkali Soil Natural Environmental Science Center (ASNESC), Northeast Forestry University, Harbin Hexing Road, Harbin 150040, China; E-Mail: ly7966@163.com; 2 Asian Natural Environmental Science Center, University of Tokyo, Nishitokyo-shi, Tokyo 188-0002, Japan; E-Mail: takano@anesc.u-tokyo.ac.jp

**Keywords:** salt stress, halophyte, *Puccinellia tenuiflora*, salt-tolerant FOX-yeast lines

## Abstract

*Puccinellia tenuiflora* is a monocotyledonous halophyte that is able to survive in extreme saline soil environments at an alkaline pH range of 9–10. In this study, we transformed full-length cDNAs of *P. tenuiflora* into *Saccharomyces cerevisiae* by using the full-length cDNA over-expressing gene-hunting system to identify novel salt-tolerance genes. In all, 32 yeast clones overexpressing *P. tenuiflora* cDNA were obtained by screening under NaCl stress conditions; of these, 31 clones showed stronger tolerance to NaCl and were amplified using polymerase chain reaction (PCR) and sequenced. Four novel genes encoding proteins with unknown function were identified; these genes had no homology with genes from higher plants. Of the four isolated genes, two that encoded proteins with two transmembrane domains showed the strongest resistance to 1.3 M NaCl. RT-PCR and northern blot analysis of *P. tenuiflora* cultured cells confirmed the endogenous NaCl-induced expression of the two proteins. Both of the proteins conferred better tolerance in yeasts to high salt, alkaline and osmotic conditions, some heavy metals and H_2_O_2_ stress. Thus, we inferred that the two novel proteins might alleviate oxidative and other stresses in *P. tenuiflora*.

## 1. Introduction

In the natural environment, plants are often subjected to salinity, drought, low temperature and other external abiotic stresses. These adverse stresses affect normal physiological and biochemical activities, which seriously hinder the normal growth of plants. Salt stress is one of the major important abiotic stress factors in nature [1,2,3]. More than 6% of the land area (about 800 million hectares) worldwide is adversely affected by salt [4]. In northeast China, the area covered by saline-alkaline soil has reached 32 million ha, and the pH of such soils is more than 9.8 [5]. Saline-alkaline erosion might accelerate the loss of arable land in the medium term, with a projected loss of up to 50% by 2050 [6]. The effect of salt stress on plant growth is a result of the combined action of many factors. These factors affect photosynthesis and various physiological and biochemical pathways, thereby retarding the growth of plants and probably even leading to their death [7,8,9,10,11]. Therefore, understanding the mechanisms by which halophytes tolerate salt stress is critical. Indeed, the discovery of genes responsible for the core signaling pathways involved in the mitigation of salt stress might allow the generation of genetically engineered plants with more favorable agricultural properties.

Loss-of-function knockout mutants and activation-tagged gain-of-function mutants are widely used for the screening and analysis of gene functions in model plants, such as *Arabidopsis thaliana* and *Oryza sativa* [12,13,14,15,16,17,18,19,20,21,22]. On the basis of these approaches, researchers have developed an alternative approach, the full-length cDNA over-expressing gene (FOX)-hunting system, which is faster and more economical. This method does not require any prior knowledge regarding the genome of interest or genetic mapping. The FOX-hunting system was first proposed as a selective screening technique for plant functional genes in 2006. In *A. thaliana*, this technique has been used to produce numerous dominant mutants, which has facilitated the elucidation of gene functions through phenotypic analysis [23]. Nakamura *et al.* [24] generated 12,000 transgenic rice strains, named as FOX-rice lines, which ectopically expressed rice full-length cDNAs under the control of the maize ubiquitin promoter and revealed that *OsGLK1* regulated chloroplast development. Nonetheless, this technique is not limited to intraspecific strategies. *Thellungiella salsuginea* is a halophilic plant with a close phylogenetic relationship with *A. thaliana*. A method similar to FOX-hunting was applied to identify salt-tolerance genes in transgenic *Arabidopsis* that overexpressed cDNAs from an expression library derived from *T. salsuginea* [25]. In all, 130 *Arabidopsis* FOX-superroot lines have been generated in bird’s-foot trefoil (*Lotus corniculatus*) for the systematic functional analysis of root genes and the selection of mutants with interesting root growth characteristics [26]. This technique is suggested to be extremely well-suited for the analysis of genes that control root length in *L. corniculatus*. Furthermore, this technique was successfully applied to identify genes that facilitated the survival of *Eichhornia crassipes* under low sulfur conditions [27]. Taken together, these data show that the FOX-hunting system is a very effective tool in plant functional gene research and that it facilitates the elucidation of the function of genes that control metabolic pathways and determine plant morphological characteristics.

*Puccinellia tenuiflora* (family, Gramineae) is a monocotyledonous halophyte distributed in the northeast region of China. Unlike other halophytes, *P. tenuiflora* can survive in extreme saline soil environments at an alkaline pH range of 9–10 [28,29]. Thus, this plant can be considered as an excellent model system for elucidating the genes involved in salt tolerance. Our previous cDNA microarray and expressed sequence tag analyses revealed many biotic and abiotic stress-induced genes that are abundantly expressed in *P. tenuiflora* [30,31]. These analyses allowed us to better understand the mechanisms of salt tolerance mechanisms in this plant. However, numerous other genes with unknown function have been reported to be differentially expressed in *P. tenuiflora* under salt stress, indicating that other genetic networks are involved in the response to stress conditions.

## 2. Results and Discussion

### 2.1. Selection of Salt-Tolerant FOX (Full-Length cDNA Over-Expressing Gene)-Yeast Lines from Yeast Full-Length P. tenuiflora cDNA Libraries

More than 1.6089 × 10^6^ full-length cDNAs were obtained, of which 1000 colonies were randomly obtained for sequencing. This revealed that over 90% of the cDNAs were full length, and the length of most of these cDNAs was distributed between 0.2 and 4.0 kb, confirming the quality of the cDNA libraries [32]. These cDNA libraries were used to transform *S. cerevisiae* strain *InVscI* and used to screen salt-tolerant FOX-yeast lines. During the first selection, 170 yeast colonies were obtained and named as NaCl-1^#^ through NaCl-170^#^. Monoclonal yeast colonies were used for secondary selection and polymerase chain reaction (PCR) identification; from these, 32 salt-tolerant FOX-yeast lines containing cDNA insertions were found.

### 2.2. Multi-Resistance and Sequence Analyses of Salt-Resistant FOX-Yeast Lines

**Figure 1:**
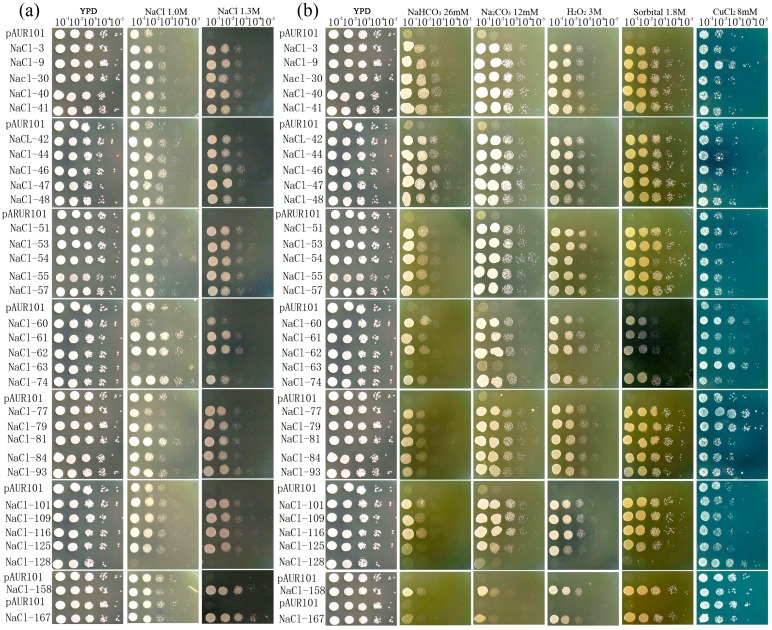
The growth of 32 salt-tolerant Fox-yeast lines yeast cells in the presence of various abiotic stresses. (**a**) The salt-tolerant Fox-yeast lines improved salt tolerance ability of yeast; (**b**) the salt-tolerant Fox-yeast lines improved other abiotic stress tolerance abilities of yeast.

Under normal growth conditions, the growth of the 32 salt-tolerant FOX-yeast lines was similar to that of the control yeast transformed with the empty pAUR101 vector (vector control, Figure 1a). When the medium was supplemented with 1 M NaCl, the growth of most salt-tolerant FOX-yeast lines were slightly improved compared with that of the vector control. When the NaCl concentration was increased to 1.3 M, the growth of the vector control was essentially blocked, whereas 31 of the 32 FOX-yeast lines continued to grow. Clones NaCl-158^#^ and NaCl-167^#^ were particularly salt-tolerant. Thus, most colonies of FOX-yeast lines showed significantly increased salt tolerance.

The relationship between the 32 FOX-yeast lines and other adverse stresses was explored by conducting multi-resistance analysis under alkaline, oxidative, osmotic and heavy metal stresses (Figure 1b). In the presence of 26 mM Na_2_CO_3_, 12 mM NaHCO_3_, 3 M H_2_O_2_ and 1.8 M sorbitol, most salt-tolerant FOX-yeast lines grew better than the vector control. The resistance profile was altered between FOX-yeast lines and the vector control after treatment with 8 mM Cu^2+^, which induced heavy metal stress (Figure 1). These data indicated that most colonies of FOX-yeast lines showed improved resistance to alkaline, oxidative and osmotic stresses. However, the FOX-yeast lines showed different resistance responses to heavy metal stress; the reason for this phenomenon was not investigated in the present study.

After multi-resistance analysis, the salt-tolerant FOX-yeast lines were subjected to molecular analysis. The sequences of primers used are listed in Table 1. Sequences were compared and analyzed using the BLAST program of the National Centre of Biotechnology Information [33]. Open reading frame (ORF) searches were conducted using ORF Finder from NCBI [34]. Full-length cDNA sequences are listed in Table 2. Thirty-two sequences were full-length cDNAs, and they had complete coding sequences with fragment lengths between 650 and 4000 bp. Inserts from the 25 FOX-yeast lines had known functions and high homology with the genes found in higher plants (such as *Arabidopsis*, rice, barley and corn). The homology of one unknown gene was similar to that of *Zea mays*, and four unknown genes were slightly homologous with those in lower organisms.

**Table 1:** Details of the primers used for polymerase chain reaction analysis.

Primer Name	Sequences (from 5' to 3')
pAUR101-FW	CCGGATCGGACTACTAGCAGCTG
pAUR101-RV	TAGGGACCTAGACTTCAGGTTGTC
pYES_2_-construct-F158	GGATCCCCGGATCGGACTATAGCAGCTG
pYES_2_-construct-R158	TCTGGATAGGGACCTAGACTTCAGGTTGTC
pYES_2_-construct-F167	GAATTCCCGGATCGGACTATAGCAGCTG
pYES_2_-construct-R167	CTCGAGTAGGGACCTAGACTTCAGGTTGTC
Put-tubulin-F	GTGTCAGCCATACTGTGCCAATC
Put-tubulin-R	TTGCTCATGCGGTCAGCAATACC
NaCl-158^#^-F	GAGCAGAGGAGCAAGATG
NaCl-158^#^-R	TTACACGGAGGACAGACAC
NaCl-167^#^-F	ACAGTTGGGAGGAGCGTC
NaCl-167^#^-R	CCACTCGATCTGCATTTCT

Underlines were restriction sites.

**Table 2:** Full-length cDNAs of *P. tenuiflora* isolated from the salt-tolerant transgenic yeast and their plant homologs.

NO.	Superfamily	Description	*E*-Value	Species and Accession
NaCl-3	RbcS superfamily	ribulose-1,5-bisphosphate carboxylase	8 × 10^−117^	*Triticum aestivum* [P00871.2]
NaCl-9	Metallothio 2 superfamily	metallothionein-like protein type 2	2 × 10^−21^	*Zea mays* [ACF85243.1]
NaCl-30	Chloroa b bind superfamily	PSI type III chlorophyll a/b-binding protein	6 × 10^−92^	*Arabidopsis thaliana* [NP_001031217.1]
NaCl-40	-	glycine-rich cell wall structural protein	3 × 10^−5^	*Oryza sativa* [EAY86471.1]
NaCl-41	NAD binding 8 superfamily	thiamin biosynthetic enzyme	0.0	Triticum urartu [EMS66450.1]
NaCl-42	-	hypothetical protein ZEAMMB73879106	-	*Zea mays* [AFW58868.1]
NaCl-44	AAI LTSS superfamily	lipid transfer protein	8 × 10^−44^	*Triticum aestivum* [ABB90546.1]
NaCl-46	PP-binding superfamily	acyl carrier protein 3	9 × 10^−63^	*Zea mays* [ACG24988.1]
NaCl-47	Plant peroxidase like superfamily	Peroxidase	1 × 10^−165^	*Glycine max* [XP_003517206.1]
NaCl-48	-	Dehydrin	3 × 10^−58^	Hordeum vulgare [CAA50499.1]
NaCl-51	DIOX-N superfamily ACC oxidase	ACC oxidase	5 × 10^−^^140^	*Glycine max* [NP_001276303.1]
NaCl-53	-	nucleic acid binding/zinc ion binding	2 × 10^−31^	*Arabidopsis thaliana* [NP_001154741.1]
NaCl-54	-	unknown	0.35	-
NaCl-55	UBQ superfamily	Polyubiquitin3	2 × 10^−167^	*Arabidopsis thaliana* [NP_851029.1]
NaCl-57	Ribosomal-S7e superfamily	40S ribosomal protein S7	2 × 10^−5^	*Cucumis sativus* [XP_004137317.1]
NaCl-60	-	neurogenic locus notch protein precursor-like	5 × 10^−89^	*Zea mays* [NP_001158958.1]
NaCl-61	Ribokinase-pfkB-like superfamily	fructokinase-2	5 × 10^−154^	*Arabidopsis thaliana* [NP_191507.1]
NaCl-62	Cupin-2 superfamily	germin-like protein	2 × 10^−^^92^	*Arabidopsis thaliana* [AAB51752.1]
NaCl-63	-	Unknown	-	-
NaCl-74	TIM-phosphate binding superfamily	glycolate oxidase	6 × 10^−72^	*Arabidopsis thaliana* [AAB80700.1]
NaCl-77	Duf1313 superfamily	EARLY flowering protein	5 × 10^−33^	*Zea mays* [ACG45265.1]
NaCl-79	DOMON superfamily	auxin-responsive protein	3 × 10^−116^	*Arabidopsis thaliana* [NP_199564.1]
NaCl-81	Glycosyltransferase-GTB-type superfamily	trehalose-6-phosphate synthase	0.0	*Oryza sativa* [NP_001063104.1]
NaCl-84	PSI-psaK superfamily	photosystem I reaction center subunit psaK	4 × 10^−52^	*Arabidopsis thaliana* [CAB53033.1]
NaCl-93	Rubredoxin-like superfamily	rubredoxin family protein	7 × 10^−62^	*Arabidopsis thaliana* [NP_001078598.1]
NaCl-101	-	Unknown	6 × 10^−11^	*Zea mays* [DAA58889.1]
NaCl-109	PsbR superfamily	PSBR (photosystem II subunit R)	7 × 10^−63^	*Oryza sativa* Japonica Group [BAC83336.1]
NaCl-116	Ntn-hydrolase superfamily	proteasome subunit beta type 4 precursor	3 × 10^−156^	*Zea mays* [ACG33740.1]
NaCl-125	-	homeodomain leucine zipper protein	2 × 10^−106^	*Zea mays* [AFW63782.1]
NaCl-128	Ribosomal-L21e superfamily	60S ribosomal protein L21	3 × 10^−38^	*Zea mays* [DAA49694.1]
NaCl-158	-	Unknown	-	-
NaCl-167	-	Unknown	-	-

### 2.3. Discovery of Two Novel Endogenous Genes of P. tenuiflora

**Figure 2:**
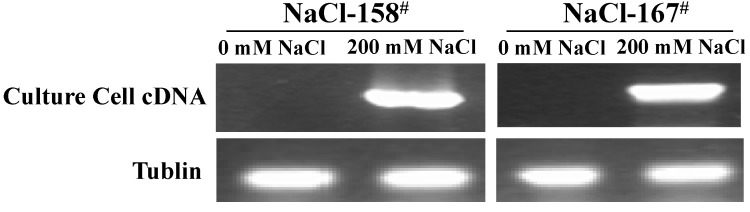
Expression of NaCl-158^#^ and NaCl-167^#^ in *P. tenuiflora* culture cells untreated and treated with 200 mM NaCl for 12 h by RT-PCR analysis.

Two of the isolated genes (NaCl-158^#^ and NaCl-167^#^) that conferred strong salt resistance had only low homology with some genes found in lower organisms, and their functions are not yet known. RT-PCR was used to amplify RNA from cultured cells of *P. tenuiflora* that were treated with 200 mM NaCl for 12 h or that were left untreated. These two genes were highly expressed in *P. tenuiflora* after treatment with 200 mM NaCl. This revealed that the two genes were strongly induced after salt stress (Figure 2). As we knew that culture cells were grown under strict sterile conditions, these exclude the possibility that we had inadvertently selected for growth of other microorganisms. These results further confirmed that the genes encoded by the cDNA inserts of NaCl-158^#^ and NaCl-167^#^ were expressed endogenously in *P. tenuiflora* and that these genes were salt inducible. Thus, we then investigated the expression patterns of the isolated genes under NaCl stress.

**Figure 3:**
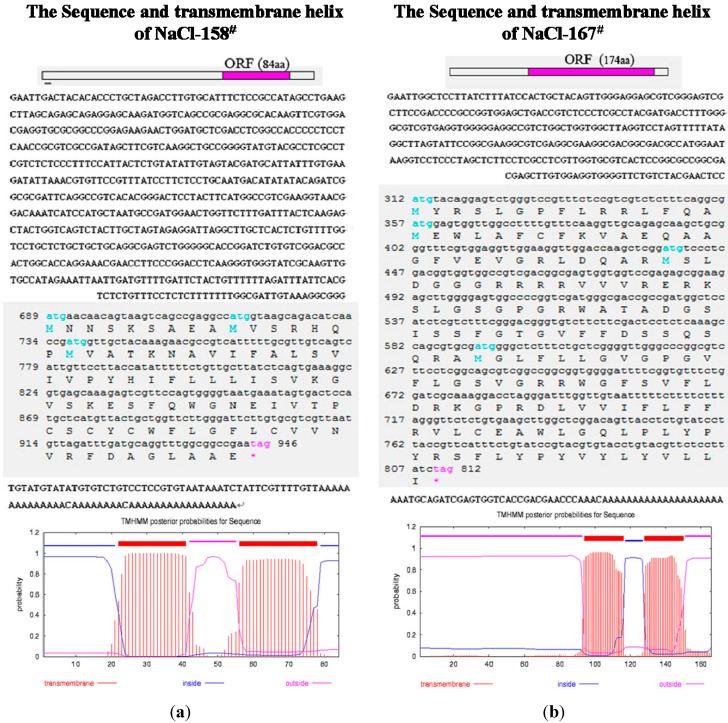
Sequence analysis of NaCl-158^#^ and NaCl-167^#^. (**a**) Open reading frame and transmembrane helix analysis of NaCl-158^#^; (**b**) open reading frame and transmembrane helix analysis of NaCl-167^#^.

The primers shown in Table 1 were used to clone the full-length sequence of NaCl-158^#^ and NaCl-167^#^. The cDNA of NaCl-158^#^ was 1036 bp in length and encoded a protein of 84 amino acids (Figure 3a). The protein had two transmembrane structural domains, as predicted by an online software TMHMM-2.0 [35] (Figure 3a). Homologous nucleotide sequences were not detected in the NCBI database by BLASTN analysis. At the amino acid level, NaCl-158^#^ showed a low level of homology with the suppressor for copper sensitivity (ScsB), a transmembrane protein isolated from *Morganella morganii* (ref|WP_004240832.1|). The cDNA of NaCl-167^#^ was 867 bp and encoded a protein of 174 amino acids (Figure 3b). The protein encoded by this gene also had two transmembrane domains (Figure 3b). Homologous nucleotide sequences were not detected in the NCBI database by BLASTN analysis. At the amino acid level, NaCl-167^#^ had low homology with a protein of unknown function, *Azospirillum brasilense Sp245* (ref|YP_005030162.1|).

### 2.4. Expression Patterns of NaCl-158^#^ and NaCl-167^#^

**Figure 4:**
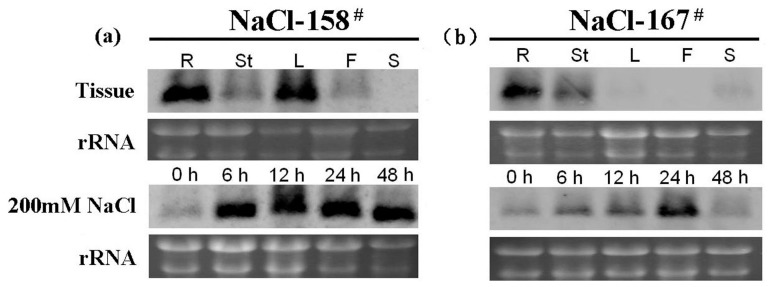
Expression analysis of NaCl-158^#^ and NaCl-167^#^ by northern blot analysis. (**a**) Analysis of the expression pattern of NaCl-158^#^ by northern blot. RNA was isolated from roots (R), stems (St), leaves (L), flowers (F) and seeds (S). The response of NaCl-158^#^ to salt stress of *P. tenuiflora* culture cells treated with 200 mM NaCl solution for the indicated times before the RNA was isolated; (**b**) Analysis of the expression pattern of NaCl-167^#^ by northern blot. RNA was isolated from roots (R), stems (St), leaves (L), flowers (F) and seeds (S). The response of NaCl-167^#^ to salt stress of *P. tenuiflora* culture cells treated with 200 mM NaCl solution for the indicated times before the RNA was isolated.

The expression patterns of NaCl-158^#^ and NaCl-167^#^ were analyzed using northern blot analysis. NaCl-158^#^ and NaCl-167^#^ were found to be constitutively expressed in *P. tenuiflora*. NaCl-158^#^ was expressed in stems and flowers, as well as in leaves and roots at high levels, but not in seeds (Figure 4a). NaCl-167^#^ was expressed in roots, stems, leaves and seeds; it was highly expressed in roots, in low levels in leaves, but not in flowers (Figure 4b).

Subsequently, the expression of NaCl-158^#^ and NaCl-167^#^ was monitored over a time course after cultured *P. tenuiflora* was treated with 200 mM NaCl. The NaCl-158^#^ transcript levels in the cultured cells increased remarkably after NaCl treatment, peaked at 6 h and remained high until 48 h (Figure 4a) On the other hand, the NaCl-167^#^ transcript levels in the cultured cells increased remarkably after NaCl treatment, peaked at 24 h and then decreased (Figure 4b). These results showed that NaCl-158^#^ and NaCl-167^#^ are constitutively expressed in *P. tenuiflora* and can be induced by NaCl stress.

### 2.5. Overexpression of NaCl-158^#^ and NaCl-167^#^ in Yeast under Various Stress Conditions

**Figure 5:**
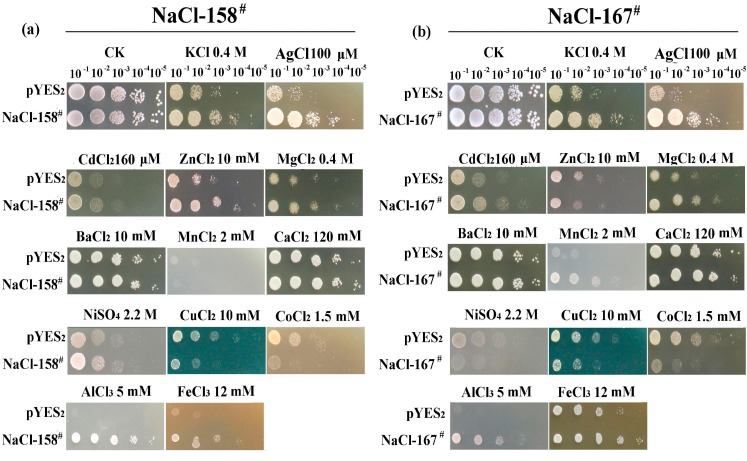
The growth of overexpressing yeast cells in the presence of various metal ions. (**a**) The growth of *pYES_2_-*NaCl-158^#^ yeast cells in the presence of various metal ions; (**b**) the growth of *pYES_2_-*NaCl-167^#^ yeast cells in the presence of various metal ions.

NaCl-158^#^ and NaCl-167^#^ are genes encoding proteins with unknown function; both proteins have two predicted transmembrane helices (Figure 3). Therefore, we speculated that these proteins might be involved in ion transport and, thus, performed metal cation-resistance analysis experiments. The two cDNAs (NaCl-158^#^ and NaCl-167^#^) were cloned into the expression vector, pYES_2_, to generate transgenic yeast. The strains were used to determine whether NaCl-158^#^ and NaCl-167^#^ could confer resistance to various metal cations. Each gene showed slightly different growth profiles in their hosts in response to different cations (Figure 5).

The growth of NaCl-158^#^ transformants was similar to that of the vector control (pYES_2_) on contrast check (CK) media, but was considerably better than that of the vector control on media containing Ag^+^, Al^3+^ and Fe^3+^; growth was slightly improved in the presence of K^+^, Zn^2+^, Ni^2+^ and Cd^2+^ and was almost the same as that of the control in the presence of Mg^2+^, Ba^2+^, Mn^2+^ and Ca^2+^. In contrast, the cells were hyper-sensitive to Cu^2+^ and Co^2+^.

The growth of NaCl-167^#^ transformants was similar to that of the vector control (pYES_2_) on CK media, but was considerably better than that of the vector control on media containing K^+^, Ag^+^, Mn^2+^, Al^3+^ and Fe^3+^; growth was slightly improved in the presence of Cd^2+^ and Mg^2+^ and was almost the same as that of control in the presence of Ba^2+^, Ca^2+^ and Zn^2+^. In contrast, the cells were slightly sensitive to Ni^2+^ and hyper-sensitive to Cu^2+^ and Co^2+^. Because of this heterogeneous response, the two transmembrane proteins could not be categorized as specific mediators of cation resistance.

**Figure 6:**
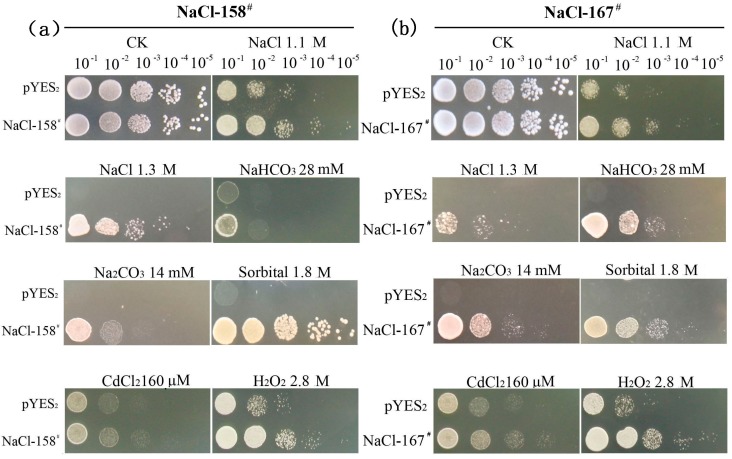
The growth of overexpressing yeast cells in the presence of various abiotic stresses. (**a**) The growth of *pYES_2_-*NaCl-158^#^ yeast cells in the presence of various abiotic stresses; (**b**) the growth of *pYES_2_-*NaCl-167^#^ yeast cells in the presence of various abiotic stresses. The pictures show the growth of *pYES_2_-*NaCl-158^#^ and *pYES_2_-*NaCl-167^#^ yeast cells in the presence of CdCl_2_ and are the same as the pictures from Figure 5.

The two yeast transformants of NaCl-158^#^ and NaCl-167^#^ were also used to confirm salt tolerance (Figure 6). Under normal conditions, the growth of NaCl-158^#^ and NaCl-167^#^ transformants was similar to that of the vector control (pYES_2_) on CK media. The growth of the vector control was blocked when the NaCl concentration reached 1.3 M, whereas the two yeast transformants continued to grow (Figure 1a. The protective effects of NaCl-158^#^ and NaCl-167^#^ in response to stress were generalized by challenging the yeast transformants to other abiotic stresses, including NaHCO_3_, Na_2_CO_3_, sorbitol and heavy metal (CdCl_2_). Both NaCl-158^#^ and NaCl-167^#^ showed enhanced growth compared to that of the vector control on all of the media. A common adverse effect of all of these treatments was the generation of oxidative stress [36,37,38,39]. The growth of the two yeast transformants was considerably better than that of the vector control on media containing H_2_O_2_. Thus, we inferred that the two genes could confer resistance to oxidative stress. This might partially explain the resistance to metal cations, since they cause oxidative stress. However, two notable exceptions were Cu^2+^ and Co^2+^, since yeast cells overexpressing NaCl-158^#^ and NaCl-167^#^ were sensitive to these ions. This might be due to the specific aspects of Cu^2+^/Co^2+^ metabolism in yeast. Yeasts are eukaryotes; their metabolic pathways are similar to those of plants. Our experimental results in yeasts provided the primary basis for performing further studies. Investigating the function of these two genes in plants is necessary to determine the mechanism of how these two genes confer stress tolerance.

## 3. Experimental Section

### 3.1. Materials

Wild-type *P. tenuiflora* plants, tissues and organs (roots, stems, leaves, flowers and seeds) were collected from the Experimental Base Alkali Soil Natural Environmental Science Center (ASNESC), Northeast Forestry University (Harbin, China). These materials were used for RNA extraction and northern blot analysis. Cultured *P. tenuiflora* cells were treated with 200 mM NaCl for 0, 6, 12, 24 and 48 h. RNA from these samples was used for RT-PCR and northern blot analyses. *S. cerevisiae* strain *InVscI* was used in the yeast transformation experiments.

### 3.2. Constructs

Construction of yeast expressing full-length cDNA libraries derived from *P. tenuiflora*: RNA samples extracted from seedlings (*P. tenuiflora*) treated with 200 mM NaCl were used. Full-length cDNA libraries of *P. tenuiflora* were constructed using the pGCAP10 vector in 2008. *Swa*I and *Not*I restriction enzyme sites were introduced at the 5' and 3' ends of each cDNA. Libraries of yeast expressing full-length cDNAs were constructed by ligating the cDNAs to the pAUR101 vector (TaKaRa, Dalian, China) after digestion with *Swa*I and *Not*I; the vectors were then transformed into *S. cerevisiae* strain *InVscI* byYeast Extract Peptone Dextrose Medium (YPD) using the LiAc/PEG yeast transformation method [40]. The transformed yeasts were cultivated on medium containing 50 mg/L abscisic acid (aba), with 3000–4000 clones on every plate. All yeast colonies were washed off the agar plates and completely resuspended. Suspensions were mixed with an equal volume of 80% glycerol, frozen in liquid nitrogen and stored at −80 °C.

Plasmid constructs: Full-length sequences of NaCl-158^#^ and NaCl-167^#^ clones were amplified from yeast DNA by using the primers, pYES_2_-construct-F158/pYES_2_-construct-R158 and pYES_2_-construct-F167/pYES_2_-construct-R167, respectively (Table 1). The amplified product was digested with restriction endonuclease and cloned into the yeast expression vector, pYES_2_ (TaKaRa), to form *pYES_2_-*NaCl-158^#^ and *pYES_2_-*NaCl-167^#^ plasmids. All products were confirmed by sequencing before use in yeast resistance tests.

### 3.3. Multiple Screening for Salt-Tolerant Transgenic Yeast Lines

Salt-tolerant yeast lines were isolated from the full-length cDNA libraries by using multiple screening and high NaCl concentration (1.3 M). Yeast library suspensions were activated, washed with sterile water 2–3 times, diluted with sterile water and coated with the solid medium, Yeast Extract Peptone Galactose Medium (YP-U), which contains 1.3 M NaCl and 2% galactose. Fifty replicates of plates containing 3000–4000 colonies/plate were interrogated to ensure full coverage of the cDNA libraries. Plates were incubated at 30 °C for 3–6 days; monoclonal FOX-yeast lines were selected and then cultured overnight in YPD liquid containing 50 mg/L aba. Cell density was adjusted on the basis of an OD_600_ of 0.5, and cells were washed twice with sterile water; 5 μL of diluted cells were spotted on 1.3 M solid NaCl YP-U medium containing 2% galactose for secondary screening. The following day, FOX-yeast lines that grew normally in 1.3 M NaCl YP-U medium were selected. Concomitantly, universal primers (pAUR101-FW and pAUR101-RV) were used to amplify DNA from the FOX-yeast lines identified in secondary screens. FOX-yeast lines that did not contain insertions were excluded from further analysis (the primers are shown in Table 1).

### 3.4. Yeast Resistance Analysis Media and Growth Conditions

All FOX-yeast lines and control strains cultured overnight were adjusted to an OD_600_ of 0.5. Ten-fold serial dilutions of all yeast were prepared (diluted over a five-log range of 10^−1^, 10^−2^, 10^−3^, 10^−4^ and 10^−5^). Five microliters of each dilution were spotted on solid YP-U medium containinga range of stress-inducing agents, including 1.0 M and 1.3 M NaCl, 26 mM NaHCO_3_, 12 mM Na_2_CO_3_, 3 M H_2_O_2_, 1.8 M sorbitol and 8 mM CuCl_2_ (containing 2% galactose), and YPD medium was used as the control. All plates were incubated at 30 °C for 3–6 days before growth was scored.

The plasmids, *pYES_2_-*NaCl-158^#^ and *pYES_2_-*NaCl-167^#^, and pYES_2_ empty vector were introduced into yeast strain *INVSCI.* Ten-fold serial dilutions of yeast were prepared, and 5 μL of each dilution were spotted on Solid yeast nitrogen base (YNB) medium (without amino acids Ura) SC-Ura medium containing a range of metal salts, including KCl, AgCl, CdCl_2_, ZnCl_2_, MgCl_2_, BaCl_2_, MnCl_2_, CaCl_2_, NiSO_4_, CuCl_2_, CoCl_2_, AlCl_3_, FeCl_3_, NaCl, NaHCO_3_, Na_2_CO_3_, sorbitol and H_2_O_2_. All of the plates were incubated at 30 °C for 3–7 days. All experiments were repeated three times.

### 3.5. Northern Blot Analysis

Total RNA was extracted using TRIzol reagent (Invitrogen, Carlsbad, CA, USA) from various organs of *P. tenuiflora* and cultured *P. tenuiflora* cells treated with 200 mM NaCl for 0, 6, 12, 24 and 48 h*.* For northern blot analysis, hybridization signals were detected using anti-DIG antibody conjugated with alkaline phosphatase (Roche, Indianapolis, IN, USA) and CDP-Star (Roche), as described by Sambrook *et al.* [41]. Signals were detected using a luminescent image analyzer (Fujifilm, LAS-4000 mini, Tokyo, Japan).

## 4. Conclusions

In all, 32 FOX-yeast lines that had stronger tolerance to salt stress, alkali stress, osmotic stress and oxidative stress were identified. These results suggest that the FOX hunting system is a rapid and effective tool for the isolation of stress tolerance genes.

In this study, two genes (NaCl-158^#^ and NaCl-167^#^) that conferred salt resistance were isolated using this method; intriguingly, these genes had only low homology with those from some lower organisms, and their functions were not yet known. In this regard, the resistance of transformed yeast to salt, alkaline conditions, osmotic stress and heavy metals indicates that the protective effect of both of the genes is directed via a common cellular pathway that is activated by all of these stresses. The oxidative stress pathway might be a prime candidate in this regard. In the future, we intend to elucidate the specific molecular mechanisms by which these genes exert their activity.
